# Eosinophilic Endomyocarditis After COVID-19 Infection in a Heart Transplant Recipient

**DOI:** 10.1016/j.jaccas.2024.102527

**Published:** 2024-09-18

**Authors:** Sara S. Inglis, Anshul R. Gupta, Francisco B. Alexandrino, Marie C. Aubry, Leslie T. Cooper, Nandan S. Anavekar, Atta Behfar, Shannon M. Dunlay, Jacob C. Jentzer, Adrian daSilva-deAbreu

**Affiliations:** a Department of Cardiovascular Medicine, Mayo Clinic, Rochester, Minnesota, USA; b Department of Internal Medicine, Mayo Clinic, Rochester, Minnesota, USA; c Department of Laboratory Medicine and Pathology, Mayo Clinic, Rochester, Minnesota, USA; d Department of Cardiovascular Medicine, Mayo Clinic, Jacksonville, Florida, USA

**Keywords:** COVID-19, eosinophilic myocarditis, heart transplantation

## Abstract

Eosinophilic myocarditis (EM) is a rare cause of heart failure, with high in-hospital mortality associated with fulminant disease. A 61-year-old female transplant recipient was diagnosed with COVID-19 after presenting with 2 days of constitutional symptoms. She developed acute heart failure from EM. After an initial response to inotropic support and corticosteroids, she had a relapse with de novo peripheral eosinophilia which responded to further eosinophilic myocarditis management and the addition of mepolizumab. Although there have been reports after COVID-19 vaccination, association with active SARS-CoV-2 infection is rare. This paper reports, for the first time, the case of a heart transplant recipient with EM after COVID-19.

## Graphical Abstract

**Figure:**
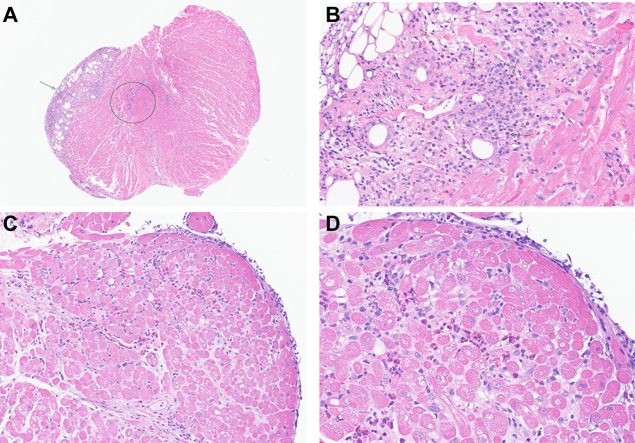

## History of Presentation

A 61-year-old female transplant recipient was diagnosed with COVID-19 after presenting with 2 days of constitutional symptoms, despite prior vaccination. Her chronic immunosuppression regimen consisted of tacrolimus 5 mg twice daily, azathioprine 100 mg daily, and prednisone 4 mg daily. She had been previously intolerant to mycophenolate due to gastrointestinal side effects. She was also on trimethoprim-sulfamethoxazole (TMP-SMX) 80 to 400 mg daily per our center’s standard protocol. Notably, although typically compliant with medical therapy, she had not been taking tacrolimus for several days due to extenuating circumstances delaying refill. Tacrolimus level was undetectable; hence, prednisone was increased to 10 mg daily until she could resume tacrolimus and reach target trough. She was discharged to continue a 5-day course of remdesivir. However, she began to develop worsening dyspnea and chest pain on exertion, prompting return to the hospital (6 days after COVID-19 diagnosis). On arrival, she was mildly tachypneic and maintaining normal oxygen saturation on room air. She had a regular tachycardia (121 beats/min) with a blood pressure of 107/78 mm Hg. On examination, decreased bibasilar breath sounds were present posteriorly. Abdomen was soft but distended. No lower extremity edema was present.

**Take-Home Messages**

• Eosinophilic myocarditis is a rare cause of heart failure associated with COVID-19, which may occur in heart transplant recipients.
• Early diagnosis and treatment of eosinophilic myocarditis is paramount to improve outcomes.

## Past Medical History

The patient’s medical history was significant for dilated cardiomyopathy and subsequent heart transplantation (HT) over 2 years prior. She was a carrier for Duchenne muscular dystrophy mutation and had no prior history of autoimmune disease or myocarditis. Her post-transplant course had been complicated by grade 2R cell-mediated rejection (CMR) 1 year after transplantation, treated with high-dose oral corticosteroids. Follow-up biopsies demonstrated 1R and 0R CMR, respectively. She had no history of pathologic antibody-mediated rejection (pAMR). Annual evaluation 8 months prior to presentation was normal.

## Differential Diagnosis

The most likely diagnosis for the patient’s clinical heart failure (HF) presentation was deemed to be acute allograft rejection and less likely acute myocarditis related to COVID-19. Cardiac allograft vasculopathy was considered unlikely given normal coronary appearance on recent annual angiographic evaluation and lack of history of pAMR or ventricular ectopy during this presentation.

## Investigations

Transthoracic echocardiogram (TTE) demonstrated new severe left ventricular (LV) dysfunction with a left ventricular ejection fraction (LVEF) of 25% to 30%. LV chamber size was normal with similar wall thickness to baseline. New mild to moderate right ventricular enlargement and moderate systolic dysfunction were noted. Tricuspid regurgitation had worsened from mild-moderate to torrential. Estimated cardiac index (CI) was 1.6 L/min/m^2^. A 3-day course of empirical intravenous methylprednisolone 1 g was initiated immediately to treat for presumed allograft rejection. After an initial improvement, she soon developed diuretic resistance with worsening hypoxemia, progressive renal dysfunction, and rising serum lactate, concerning for cardiogenic shock. Right heart catheterization and endomyocardial biopsy (EMB) were expedited, demonstrating increased biventricular filling pressures and a CI of 1.65 L/min/m^2^.

## Management

**Figure 1:**
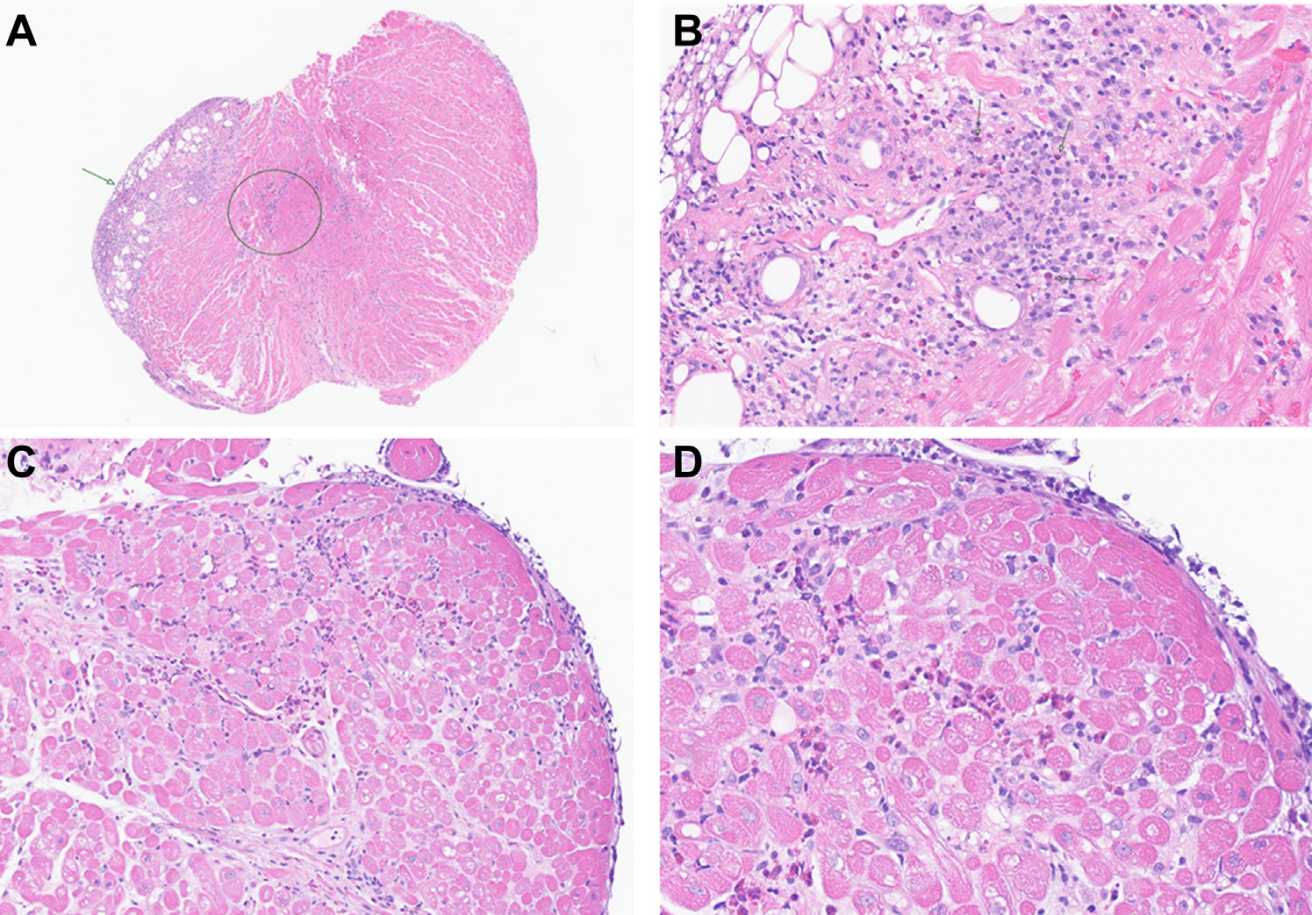
Endomyocardial Biopsies (A) Endocardial inflammation (arrow) and International Society for Heart and Lung Transplantation 2004 grade 1R cell-mediated rejection (circle) (H&E, 40×). (B) High power visualization of severe inflammation rich in eosinophils (arrows) (H&E, 200×). (C) Subsequent biopsy showing endomyocardial inflammation (H&E, 100×). (D) High power image of inflammation composed of eosinophils (H&E, 400×). H&E = hematoxylin and eosin.

She was briefly initiated on milrinone, followed by clevidipine because blood pressure increased, with normalization of hemodynamics and no escalation to temporary mechanical circulatory support. EMB demonstrated severe inflammation with marked numbers of eosinophils, predominantly involving the endocardium, consistent with eosinophilic endomyocarditis. Appearance was not in keeping with typical cellular rejection, which in severe cases may be rich in eosinophils, but would also be accompanied by more severe myocyte damage and a brisk lymphocytic infiltrate, features which were not present. Mild rejection was noted (1R CMR, pAMR 0) characterized by a focus of perivascular lymphoid infiltrate, distinct from the eosinophilic endomyocarditis (Figures 1A and 1B).

Review of the patient’s medications revealed TMP-SMX and azathioprine as potential culprits for eosinophilic infiltration; however, they were considered unlikely given longstanding exposure. Stool studies for ova and parasites, Strongyloides culture, and Toxocara antibodies were negative. In addition, review of allograft donor history from United Network for Organ Sharing revealed no history of parasitic infections or travel to endemic regions.

**Figure 2:**
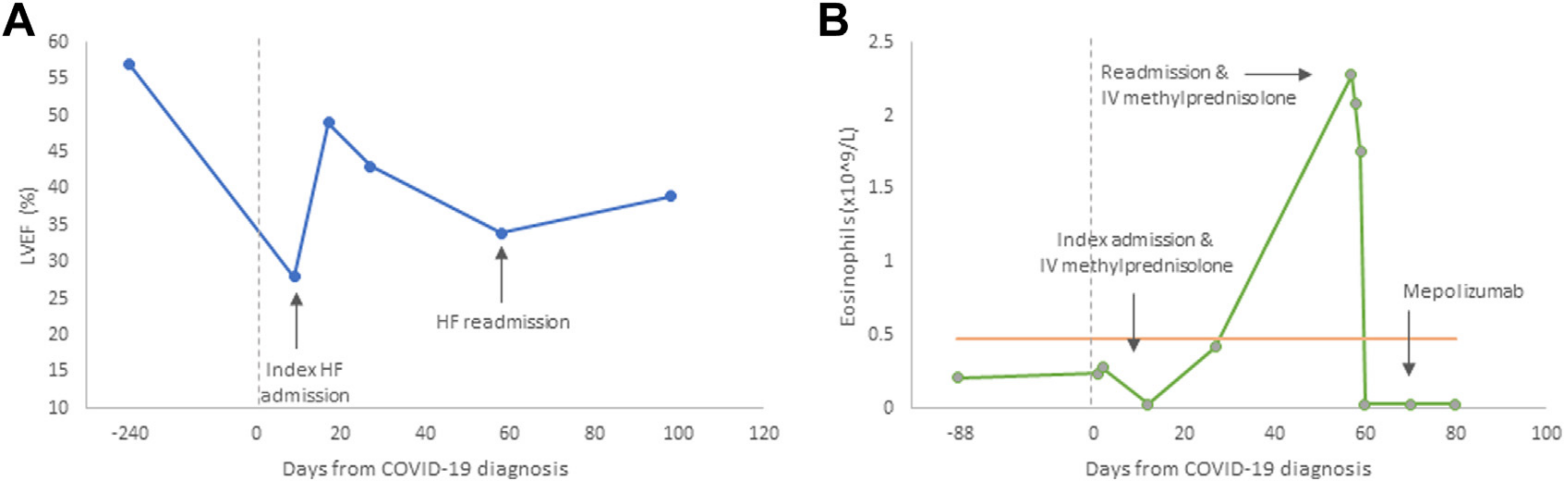
Trends in LVEF and Blood Eosinophils From Time of COVID-19 Diagnosis (A) Normal cardiac function demonstrated prior to COVID-19 diagnosis, followed by steep decline in the setting of eosinophilic myocarditis. Initial improvement after treatment, followed by subsequent decline at readmission with recurrent cardiogenic shock. (B) Peripheral blood eosinophil levels. Baseline eosinophils within normal limits, acute rise at readmission, and improvement after reinitiation eosinophilic myocarditis treatment. Orange line represents the upper limit of normal. HF = heart failure; IV = intravenous; LVEF = left ventricular ejection fraction.

The patient completed the 3-day course of daily intravenous methylprednisolone 1 g and was weaned off inotropes on day 4 of hospitalization. She underwent cardiac magnetic resonance on day 10, revealing an enlarged LV chamber size with normal wall thickness. Mild basal hypokinesis was present with an LVEF of 49%. She was discharged from the hospital on day 13. She continued a corticosteroid taper, reaching her baseline prednisone dose (4 mg daily) 17 days after dismissal. Three weeks later, she again developed progressive HF symptoms and was readmitted. Repeat TTE revealed worsening LVEF to 34% (Figure 2A), with moderate to severe tricuspid regurgitation and right ventricular dysfunction. Estimated CI was once again low at 1.71 L/min/m^2^. Milrinone and intravenous methylprednisolone were reinitiated, with a plan for more prolonged corticosteroid taper. Interestingly, although initially absent, peripheral eosinophils were elevated on readmission (2.28 × 10^9^/L) (Figure 2B). Repeat EMB revealed stable grade 1R CMR, negative for pAMR, and eosinophilic endomyocardial disease (Figures 1C and 1D).

**Figure 3:**
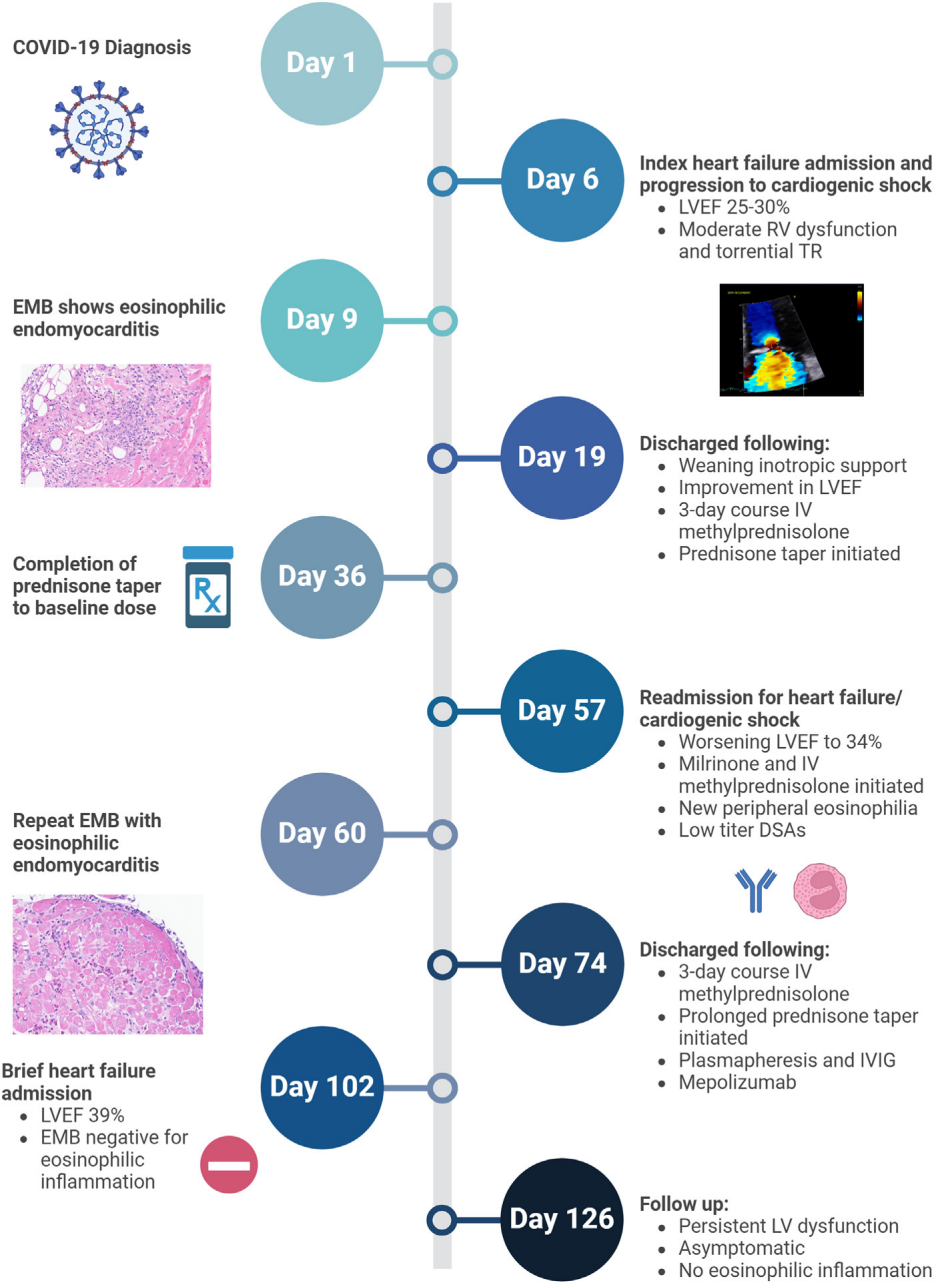
Time Line Summarizing Clinical Course After COVID-19 Diagnosis Created with BioRender.com. DSA = donor-specific antibodies; EMB = endomyocardial biopsy; IV = intravenous; IVIG = intravenous immunoglobulin; LV = left ventricular; LVEF = left ventricular ejection fraction; RV = right ventricular; TR = tricuspid regurgitation.

The patient underwent a bone marrow assessment which was unremarkable. Donor-specific antibodies (DSAs) were mildly elevated (Table 1). In addition to acute management for recurrent endomyocardial eosinophilic infiltration with methylprednisolone, she underwent plasmapheresis for 3 days followed by intravenous immunoglobulin therapy. In light of new peripheral eosinophilia and recurrent eosinophilic endomyocarditis, mepolizumab (anti-interleukin [IL]-5 monoclonal antibody) was also initiated. Repeat TTE revealed an LVEF of 42%, and she was discharged home soon after. A summary of her clinical course from COVID-19 diagnosis is outlined in Figure 3.

**Table 1:** DSAs

DSA	Time From COVID-19 Diagnosis				
	8 Months Prior (Annual Evaluation)	57 Days (HF Readmission)	65 Days	67 Days	69 Days (Postplasmapheresis)
A∗02:01	518	650	1,809	894	<500
B∗44:03	<500	3,202	5,625	3,999	1,903
B∗57:01	<500	782	2,047	1,341	<500

DSA = donor-specific antibody; HF = heart failure.

## Outcome and Follow-Up

The patient was readmitted briefly for a mild decompensation of HF 1 month after discharge from her second HF hospitalization. Repeat EMB demonstrated ongoing grade 1R CMR with no pAMR and absence of eosinophils. One month later, LVEF remained low but without HF symptomatology and EMB remained negative for further eosinophilic infiltration.

## Discussion

Eosinophilic myocarditis (EM) has been rarely reported after HT and is considered unusual due to the chronic immunosuppression which is expected to suppress eosinophilic inflammation. Although more commonly lymphocytic, EM has been reported after COVID-19 infection or vaccination, and this was deemed to be the probable etiology in this case.^1^, ^2^, ^3^

EM is a rare cause of HF, with variable clinical presentation and high in-hospital mortality associated with fulminant disease. Recovery is common after circulatory support and systemic corticosteroids, highlighting the importance of early diagnosis and treatment.^1^^,^^4^ EM may be secondary to parasitic infections, hypersensitivity reactions, autoimmune diseases, malignancies, or idiopathic.^1^^,^^4^ Hypersensitivity reactions represent a common cause of EM and may occur without peripheral eosinophilia in the absence of drug rash with eosinophilia and systemic symptoms.^4^ Overall, peripheral eosinophilia may be absent in up to 25% of EM cases.^4^ Although there have been several documented reports of EM associated with COVID-19 vaccination, association with active infection is rare and has not been described in HT recipients.^1^, ^2^, ^3^^,^^5^^,^^6^

In the present case, inflammatory cytokines important for eosinophil maturation (ie, IL-3, IL-5, granulocyte-macrophage colony-stimulating factor) may have been unsuppressed despite ongoing azathioprine use, which is cause for speculation. Perhaps under certain circumstances (eg, when receiving azathioprine and/or TMP-SMX treatment), the response to COVID-19 injury may be directed toward eosinophil dominance. Adding to perplexity in this case was the new development of low titer DSAs. Eosinophils have historically been implicated in allograft rejection. However, the present case lacks convincing histologic evidence, without severe myocyte damage and lymphocytic infiltrate. In the absence of a compelling alternative, COVID-19 was considered the likely inciting culprit due to the proximity of diagnosis, in tandem with previously documented cases in native hearts.

## Conclusions

To our knowledge, this is the first report of fulminant eosinophilic endomyocarditis after COVID-19 infection in an HT recipient, who was initially treated with inotropic therapy and high-dose corticosteroids. Importantly, absence of peripheral eosinophilia does not preclude this diagnosis and early diagnosis/treatment is paramount. Interestingly, this patient also developed early recurrence with de novo peripheral eosinophilia, which abated with the addition of mepolizumab.

## Funding Support and Author Disclosures

The authors have reported that they have no relationships relevant to the contents of this paper to disclose.
